# Identification of prognostic immune-related lncRNA signature predicting the overall survival for colorectal cancer

**DOI:** 10.1038/s41598-023-28305-9

**Published:** 2023-01-24

**Authors:** Jianxin Li, Ting Han, Xin Wang, Yinchun Wang, Xuan Chen, Wangsheng Chen, Qingqiang Yang

**Affiliations:** Department of General Surgery (Gastrointestinal Surgery), The Affiliated Hospital of Southwest Medical University, 25 Taiping Street, Luzhou, 646000 Sichuan People’s Republic of China

**Keywords:** Cancer genomics, Cancer models, Biomarkers, Gastroenterology

## Abstract

Long non-coding RNA (lncRNA) is an important regulator of gene expression and serves a fundamental role in immune regulation. The present study aimed to develop a novel immune-related lncRNA signature to assess the prognosis of patients with colorectal cancer (CRC). Transcriptome data and clinical information of patients with CRC were downloaded from The Cancer Genome Atlas (TCGA) and UCSC Xena platforms. Immune-related mRNAs were extracted from the Molecular Signatures Database (MSigDB), and the immune-related lncRNAs were identified based on correlation analysis. Then, univariate, Lasso and multivariate Cox regression were applied to construct an immune-related lncRNA signature, and CRC patients were divided into high- and low-risk groups according to the median risk score. Finally, we evaluated the signature from the perspectives of clinical outcome, clinicopathological parameters, tumor-infiltrating immune cells (TIICs), immune status, tumor mutation burden (TMB) and immunotherapy responsiveness. In total, 272 immune-related lncRNAs were identified, five of which were applied to construct an immune-related lncRNA signature. The signature divided patients with CRC into low- and high-risk groups, the prognosis of patients in the high-risk group were significantly poorer than those in low-risk group, and the results were further confirmed in external validation cohort. Furthermore, the high-risk group showed aggressive clinicopathological characteristics, specific TIIC and immune function status, and low sensitivity to immunotherapy. The immune-related lncRNA signature could be exploited as a promising biomarker for predicting the prognosis and immune status of patients with CRC.

## Introduction

Colorectal cancer (CRC) is among the most diagnosed malignancies in digestive system and ranks second in terms of cancer-related death^1^. Every year, more than 1.8 and 0.9 million estimated new CRC cases and deaths worldwide^2^. Recently, with the development of research, the pathophysiological and molecular mechanisms underlying the onset, invasion and metastasis of CRC have been extensively explored, and significant progress has been made in the diagnosis and treatment of CRC. However, CRC remains a severe health threat to humans because of the deficiency of effective biomarkers for diagnosis, especially in the early stage, and the prognosis of CRC patients is still poor. The 5-year survival rate for patients diagnosed with early stage is about 90%, while the 5-year survival rate sharply declines to 12% for patients diagnosed with advanced stage^3^. Considering the current situation of the diagnosis and treatment of CRC, it is imperative to seek more effective assessment models to evaluate the prognosis of CRC patients and provide optimized strategies for clinical CRC treatment.

In the past few years, accumulating studies have indicated that disorder of the immune response in the tumor microenvironment (TME) was significantly correlated with cancer development^4^. Immune cells are able to suppress the proliferation, invasion, and metastasis of cancer cells in the TME by perturbing the molecular signal pathway^5^. Nevertheless, some cancer cells also can inhibit the immune responses and create environments suitable for tumor growth, while these effects could be reversed by immunotherapy^6^. Therefore, immunotherapy provides an unprecedented opportunity for the effective treatment of cancers because of the crucial regulatory role of the immune cells in the TME^7^. Long non-coding RNAs (lncRNAs) are defined as transcripts with nucleotides more than 200 in length and have no ability to encode protein^8^. Previous studies have indicated that lncRNA can regulate gene expression at multiple levels, such as epigenetic, transcriptional or post-transcriptional regulation, and dysfunction of lncRNA is closely associated with the progression of multiple diseases, including malignancies^9–11^. Moreover, recent studies also indicated that lncRNA plays a significant regulatory role in different phases of tumor immunity, and several lncRNAs have been identified as potential biomarkers for many cancers^12,13^. For example, lncRNA SNHG1 inhibits the immune escape of breast cancer cells by suppressing the differentiation of Treg cells^14^. Similarly, NKILA regulates cytotoxic T lymphocyte sensitivity to activation-induced cell death by inhibiting NF-κB activity, thereby promoting tumor immune evasion of breast cancer^15^. These findings indicated that immune-related lncRNAs serve as potential tumor biomarkers and deserve more attention.

Previous research has indicated that multiple genes combinations is better than single gene in terms of the efficiency of a diagnostic model for tumors^16^. Recently, several studies have focused on developing a prognostic signature based on immune-related lncRNA and found that the signature could serve as a prognostic biomarker in human CRC^17,18^. However, most of these signatures are comprised of lncRNA pairs, which contain too much component. Besides, external validation was needed to improve the universal applicability of these developed signatures. Furthermore, the correlations between the developed signatures and immune cell phenotypes should be illustrated to identify which group of CRC patients is more likely to benefit from immunotherapy. Thus, we analyzed the entire RNA-sequencing profile data obtained from The Cancer Genome Atlas (TCGA) and developed a five-lncRNA immune-related signature for the prediction of prognosis in patients with CRC. In our study, we further investigated the predictive abilities of this signature in human CRC from the perspectives of clinicopathological parameters, tumor-infiltrating immune cells (TIICs), immune status, tumor mutation burden (TMB), and immunotherapy responsiveness. Importantly, the robustness of the immune-related lncRNA signature was validated in different populations from Gene Expression Omnibus (GEO) database.

## Materials and methods

### Data acquisition and processing

The entire RNA-sequencing profile data of colon adenocarcinoma (COAD) and rectal adenocarcinoma (READ) in the format of Fragments Per Kilobase Million (FPKM) were downloaded from TCGA (https://gdc.cancer.gov/). Then, the protein-coding genes and lncRNAs were classified by using the gene annotation function of the GENCODE project (https://www.gencodegenes.org/)^19^. In addition, the patient’s clinical parameters, including survival time, survival status, age, gender, American Joint Committee on Cancer (AJCC) stage, microsatellite instability, histological type and venous invasion status, were downloaded from the UCSC Xena (http://xena.ucsc.edu/) platform^20^. The gene expression profile matrix file and corresponding clinical information of GSE17536 were downloaded from GEO database (https://www.ncbi.nlm.nih.gov/geo/) and used as external validation cohort^21^. There is no need for approval by the ethics committee because all the data involved in the present study were obtained from the public database.

### Identification of immune-related lncRNAs

The immune-related mRNAs were extracted from immunomodulatory gene datasets IMMUNE RESPONSE (M19817) and IMMUNE SYSTEM PROCESS (M13664) based on the Molecular Signatures Database (MSigDB, https://www.gsea-msigdb.org/gsea/msigdb/)^22^. Then, the correlation between lncRNAs and immune-related mRNAs was calculated using the Pearson correlation analysis. lncRNAs with Pearson correlation coefficient > 0.5 and *P* < 0.001, and meanwhile interacted with more than two immune-related mRNAs were identified as immune-related lncRNAs and selected for further analysis. To enhance the reliability of our study, the entire expression cohort of immune-related lncRNAs was randomly divided into the training cohort and the test cohort by the “caret” package in R.

### Construction of an immune-related lncRNA signature associated with prognosis

To identify the survival-related lncRNAs, the prognostic significance of immune-related lncRNAs was analyzed by univariate Cox regression. Those immune-related lncRNAs with *P* < 0.01 in univariate analysis were included in the LASSO regression analysis to minimize the over-fitting. Then, we conducted a multivariate Cox analysis to construct the risk signature calculated as: Risk score = Ʃ (βi × Expi)^23^. Where the βi and Expi represented the coefficient index and the gene expression level, respectively. Patients were divided into high- and low-risk groups according to the median value of the risk score in the training cohort.

### Prediction analysis of risk score signature

To evaluate the prognostic value of the immune-related lncRNA signature in human CRC, we depicted the survival curve between high- and low-risk groups by using the Kaplan–Meier method with a log-rank test. Additionally, we applied the time-dependent receiver operating characteristic (ROC) curve and area under the ROC curve (AUC) to estimate the diagnostic efficacies. Similarly, we evaluate the prognostic value of this signature in the test cohort and the entire TCGA cohort to verify the reliability of the signature. To further confirm the clinical value of the immune-related lncRNA signature, we assessed the associations between the risk signature and clinical parameters. Finally, we compared the prognostic value of clinical parameters and risk score to determine whether the signature can be used as an independent prognostic factor.

To further determine whether the immune-related lncRNA signature had a similar prognostic value in different populations, its prognostic capability was validated using external cohort.

### Co-expression analysis and enrichment analysis

To further investigate the biological function of the immune-related lncRNA signature, we constructed a co-expressed lncRNA-mRNA network by using Pearson correlation based on the correlation coefficient threshold > 0.5 and the corresponding *P* < 0.001. After that, we used the “clusterProfiler” package in R to carry out Gene Ontology (GO) enrichment analysis, including biological process (BP), cell components (CC) and molecular function (MF) for the co-expressed network. The Kyoto Encyclopedia of Genes and Genomes (KEGG) enrichment analysis is also carried out by the same tool^24^.

### Construction of predictive nomogram

By using “rms”, “foreign”, and “survival” packages in R, we constructed a nomogram consisting of relevant clinical parameters and risk score based on the multivariate Cox regression analysis to predict survival for the patients with CRC. The calibration curve and ROC curves were applied to investigate the calibration and the discrimination of the nomogram^25^.

### Immune characteristics analysis

To determine the correlation between the risk score and TIIC characteristics, we applied the QUANTISEQ algorithm to calculate the abundances of distinct TIIC among these samples from the TCGA project^26^. Besides, we also analyzed the differences in TIIC abundances and immune-related biological functions between low- and high-risk groups by applying single sample GSEA (ssGSEA) algorithm^27^.

### Tumor mutation burden in different subgroups

To identify the TMB of low-risk group and high-risk group, we obtained the information of somatic mutation profiles of CRC from TCGA sample. The differences in TMB between low- and high-risk groups were analyzed by performing Wilcoxon signed-rank test. Then, we analyzed and demonstrated the gene mutation patterns and frequencies in different subgroups by using the R package “maftool”^28^.

### Tumor immune dysfunction and exclusion analysis

Tumor immune dysfunction and exclusion (TIDE, http://tide.dfci.harvard.edu/) is a computational method to predict the clinical responses to immunotherapy and the function of T-cells in TME^29^. In this study, we applied the TIDE algorithm to predict the individual response to immunotherapy and compare the difference in TIDE scores between low- and high-risk groups.

### Statistical analysis

The statistical analyses were performed via the R software (v 4.0.2, https://www.r-project.org/). The correlations of gene expression were evaluated by Pearson’s correlation coefficient. Cox regression and LASSO regression were used to evaluate the prognostic significance of the immune-related lncRNA signature and clinicopathological data. Chi-square test was used to evaluate the differences in clinicopathological features between two groups of patients. Fisher’s exact test was used while the sample size was less than five. Kaplan–Meier curves and log-rank tests were utilized to evaluate the survival data. The predictive accuracy of the risk model was determined by ROC curves. Wilcoxon test was mainly utilized for comparing two groups, and Kruskal–Wallis test was used for more than two groups. Fisher’s exact test was applied to evaluate the significant GO terms and KEGG enrichment pathways. *P*-value < 0.05 was considered as statistically significant.

## Results

### Identification of immune-related lncRNAs

In total, 14,142 lncRNAs and their expression profiles were obtained from the TCGA database, and the list of 331 immune-related mRNAs was identified from the MSigDB. Subsequently, Pearson correlation analysis between the lncRNAs and immune-related mRNAs was performed, and 272 lncRNAs with the characteristics that correlation coefficient > 0.5, *P* < 0.001, and meanwhile interacted with more than two immune-related mRNAs were considered as immune-related lncRNAs. The entire expression profile of the immune-related lncRNAs was randomly divided into the training cohort and the test cohort using the “caret” package in R. As shown in Table 1, there were no significant differences in clinical parameters between the training cohort and the test cohort. Then, the expression profiles of the 272 immune-related lncRNAs in the training cohort were selected for subsequent study.

**Table 1:** Clinical features of the patients with CRC in each cohort.

Variables	Training cohort (n = 253)	Test cohort (n = 253)	*P*	Entire TCGA cohort (n = 506)
Age (years)				
≤ 65	120	105	0.2104	225
> 65	133	148		281
Gender				
Female	105	125	0.0898	230
Male	148	128		276
Survival status				
Alive	209	206	0.8169	415
Dead	44	47		91
Tumor invasion (T)				
T1	7	8	0.9783	15
T2	45	46		91
T3	174	170		344
T4	27	29		56
Lymph node (N)				
N0	150	145	0.7674	295
N1	63	61		124
N2	40	46		86
Unknown	0	1		1
Metastasis (M)				
M0	212	212	0.5926	424
M1	38	35		73
Unknown	3	6		9
Tumor stage				
Stage I	44	47	0.6873	91
Stage II	101	90		191
Stage III	67	75		142
Stage IV	38	35		73
Unknown	3	6		9
Microsatellite instability				
MSS	168	170	0.5302	338
MSI-L	41	43		84
MSI-H	36	37		73
Unknown	8	3		11
Histological type				
Adenocarcinoma	217	222	0.3081	439
Mucinous adenocarcinoma	31	30		61
Unknown	5	1		6
Venous invasion				
Yes	60	50	0.5593	110
No	158	166		324
Unknown	35	37		72

### Construction of the immune-related lncRNA signature

**Figure 1:**
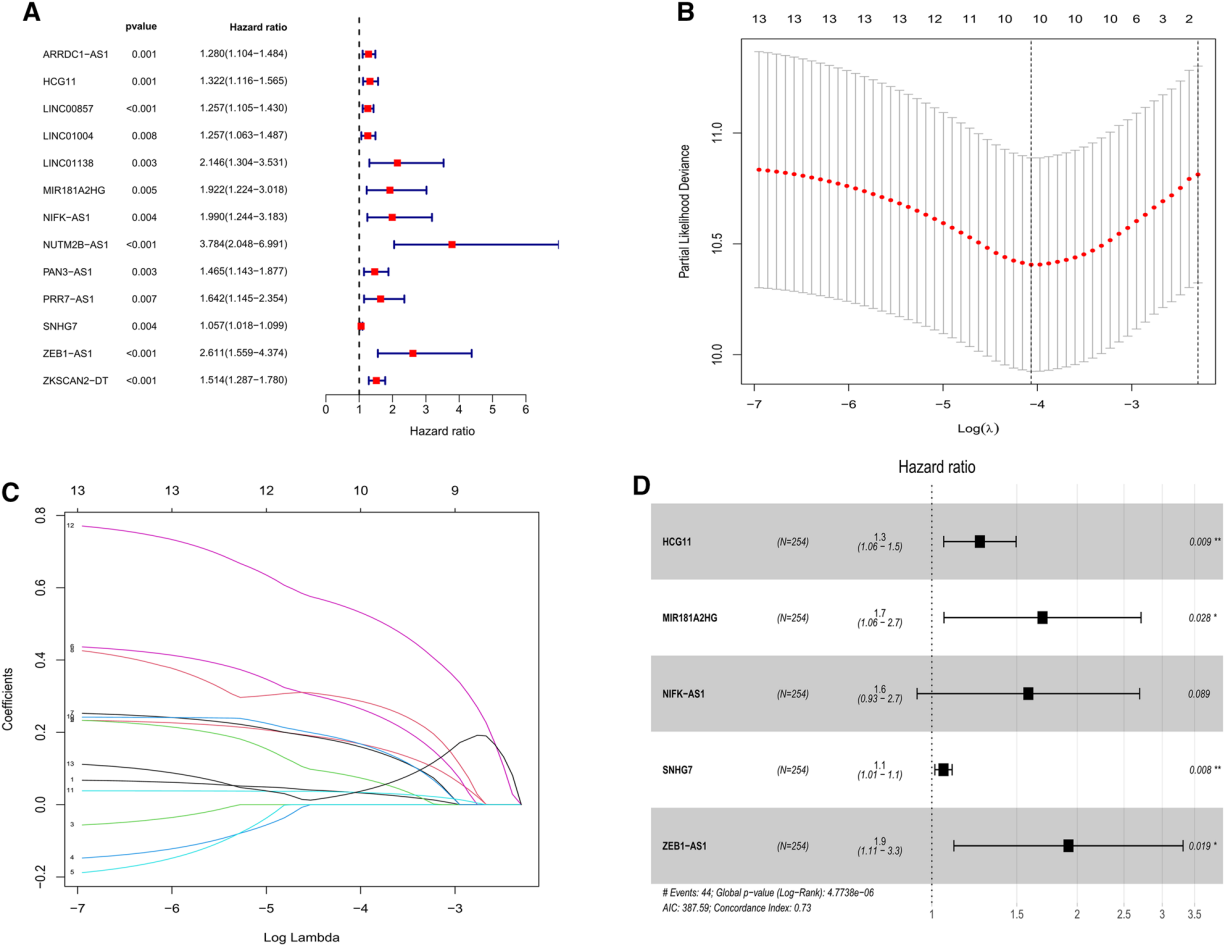
Construction of immune-related lncRNA signature associated with prognosis of patients with CRC. (**A**) Univariate Cox regression models identified 13 immune-related lncRNAs associated with overall survival. (**B,C**) Lasso regression analysis identified 10 immune-related lncRNAs. (**D**) Multivariate Cox regression models identified five immune-related lncRNAs to construct the prognostic signature.

We carried out univariate Cox regression model to assess the prognostic significance of the 272 immune-related lncRNAs in the training cohort and found that 13 lncRNAs were significantly associated with the overall survival (OS) of CRC patients (*P* < 0.01, Fig. 1A). Subsequently, we performed LASSO regression with a tenfold cross-validation on these lncRNAs in order to avoid over-fitting the prognostic signature. As a result, ten genes remained from 13 significant prognosis-related lncRNAs in the univariate Cox regression model by using LASSO regression model (Fig. 1B,C). Then, five lncRNAs were identified as independent prognostic factors by multivariate Cox regression model, and its regression coefficients (β) were also determined for further analysis (Fig. 1D, Table 2). Finally, based on the expression levels of these five lncRNAs, the risk score of each patient was calculated as follow: riskScore = (0.22928 × Exp_*HCG11*_) + (0.52785 × Exp_*MIR181A2HG*_) + (0.46034 × Exp_*NIFK-AS1*_) + (0.05573 × Exp_*SNHG7*_) + (0.65167 × Exp_*ZEB1-AS1*_).

**Table 2:** Multivariate Cox results of lncRNAs based on TCGA data.

lncRNA	Coefficient (β)	HR	95% CI of HR
HCG11	0.22928	1.258	1.058–1.494
MIR181A2HG	0.52785	1.695	1.059–2.712
NIFK-AS1	0.46033	1.585	0.932–2.693
SNHG7	0.05573	1.057	1.015–1.102
ZEB1-AS1	0.65167	1.919	1.111–3.314

*HR* hazard ratio, *CI* confidence interval.

### The prognostic influence of the established signature in TCGA

**Figure 2:**
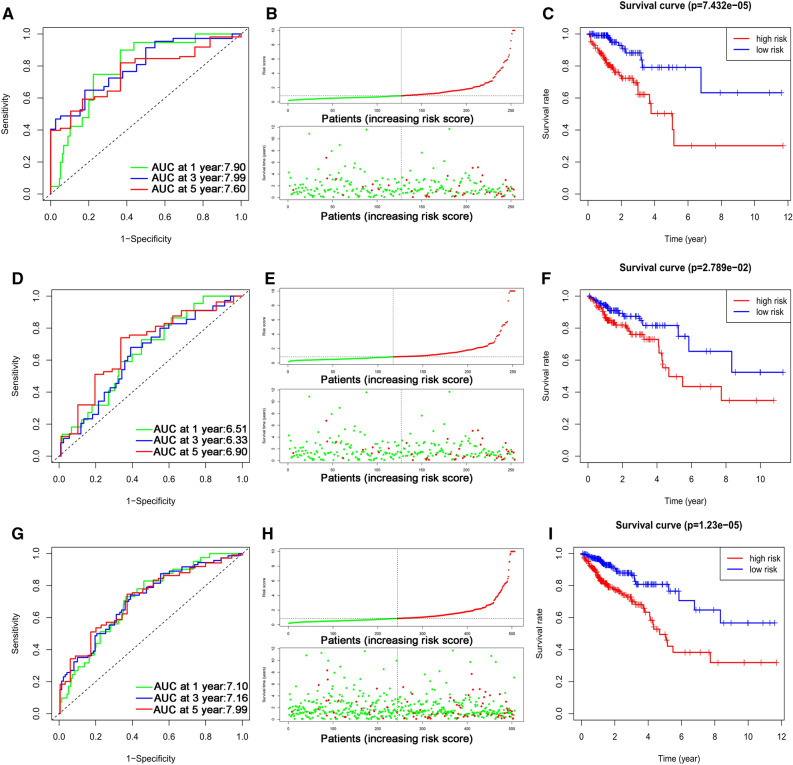
The prognostic value of the immune-related lncRNA signature. (**A**) Time-dependent ROC curve analysis, (**B**) risk score and vital status, and (**C**) Kaplan–Meier survival curve of the immune-related lncRNA signature in the training cohort. (**D**) Time-dependent ROC curve analysis, (**E**) risk score and vital status, and (**F**) Kaplan–Meier survival curve of the immune-related lncRNA signature in the test cohort. (**G**) Time-dependent ROC curve analysis, (**H**) risk score and vital status, and (**I**) Kaplan–Meier survival curve of the immune-related lncRNA signature in the entire TCGA cohort.

The CRC patients were divided into a low-risk group (n = 253) and a high-risk group (n = 253) according to the median risk score. We assessed the predictive performance of the five-lncRNA immune signature by time-dependent ROC curves and found that the AUC for 1-, 3-, and 5-year OS were 0.790, 0.799, and 0.760, respectively (Fig. 2A). Vital status analysis showed that the patient’s mortality rate increased with the increase of risk score (Fig. 2B). The Kaplan–Meier log-rank test revealed that patients in the high-risk group suffered a shorter survival time than those in the low-risk group (*P* < 0.05, Fig. 2C). Then, we applied the test cohort to validate the predictive ability of the five-lncRNAs immune signature. Time-dependent ROC curves showed that the AUC for 1-, 3-, and 5-year OS were 0.651, 0.633, and 0.690, respectively (Fig. 2D). Vital status analysis showed that the patient’s mortality rate increased with the increase of risk score (Fig. 2E). Kaplan–Meier curves showed that patients in the high-risk group suffered a shorter survival time than those in the low-risk group (*P* < 0.05, Fig. 2F). Similarly, we verified the predictive ability of this immune-related lncRNA signature in the entire TCGA cohort and found that the AUC for 1-, 3-, and 5-year OS were 0.710, 0.716, and 0.799, respectively (Fig. 2G). Vital status analysis showed that the patient mortality rate increased with the increase of risk score (Fig. 2H). Kaplan–Meier curves showed that patients in the high-risk group suffered a shorter survival time than those in the low-risk group (*P* < 0.05, Fig. 2I).

**Figure 3:**
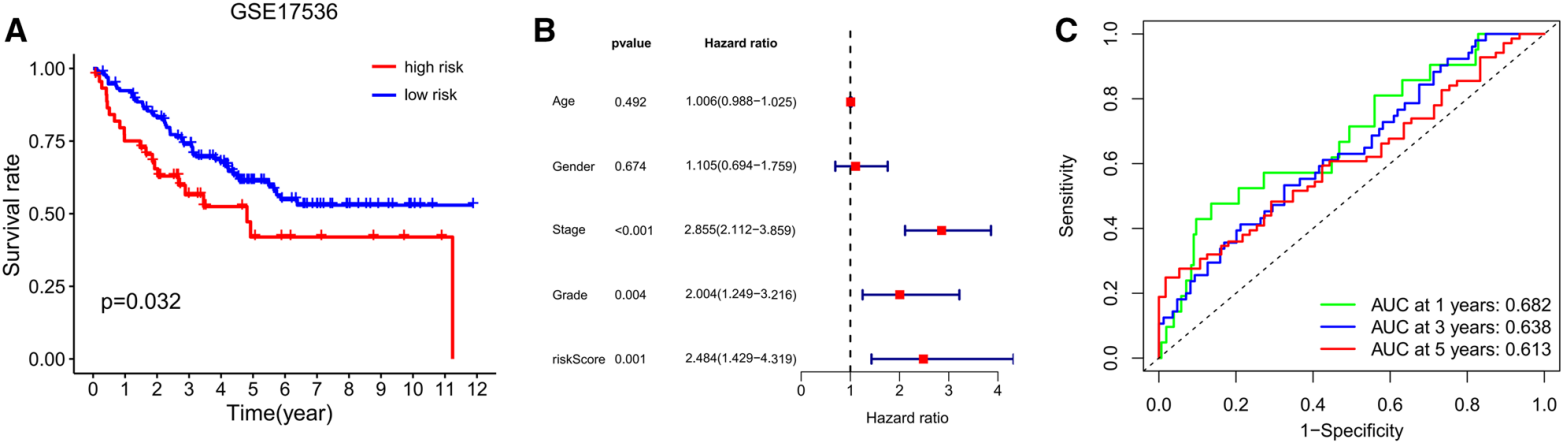
Validation of the immune-related lncRNA signature in external test cohort. (**A**) Kaplan–Meier survival curve of patients with CRC in high- and low-risk groups in GSE17536 cohort. (**B**) Cox analysis of prognostic factors in GSE17536 cohort. (**C**) Time-dependent ROC curve for predicting 1-, 3- and 5-year survival in GSE17536 cohort.

To further validate the robustness of the immune-related lncRNA signature in survival prediction in different populations, GSE17536 dataset from GEO database was set as external test cohorts. As shown in Fig. 3A, we found that the OS of patients with CRC in high-risk group were significantly lower than those in low-risk group (*P* = 0.032). Besides, cox regression analysis also found that the risk score serves as a risk factor for the prognosis of CRC patients (*P* = 0.001, HR 2.484, 95% CI [1.429–4.319]) (Fig. 3B). Correspondingly, the 1-, 3-, and 5-year AUC values for the external cohort in predicting prognosis was 0.682, 0.638, and 0.613, respectively (Fig. 3C). These findings above suggested that the immune-related lncRNA signature was competent for predicting the prognosis of CRC patients.

### Clinical values of the immune-related lncRNA signature

**Figure 4:**
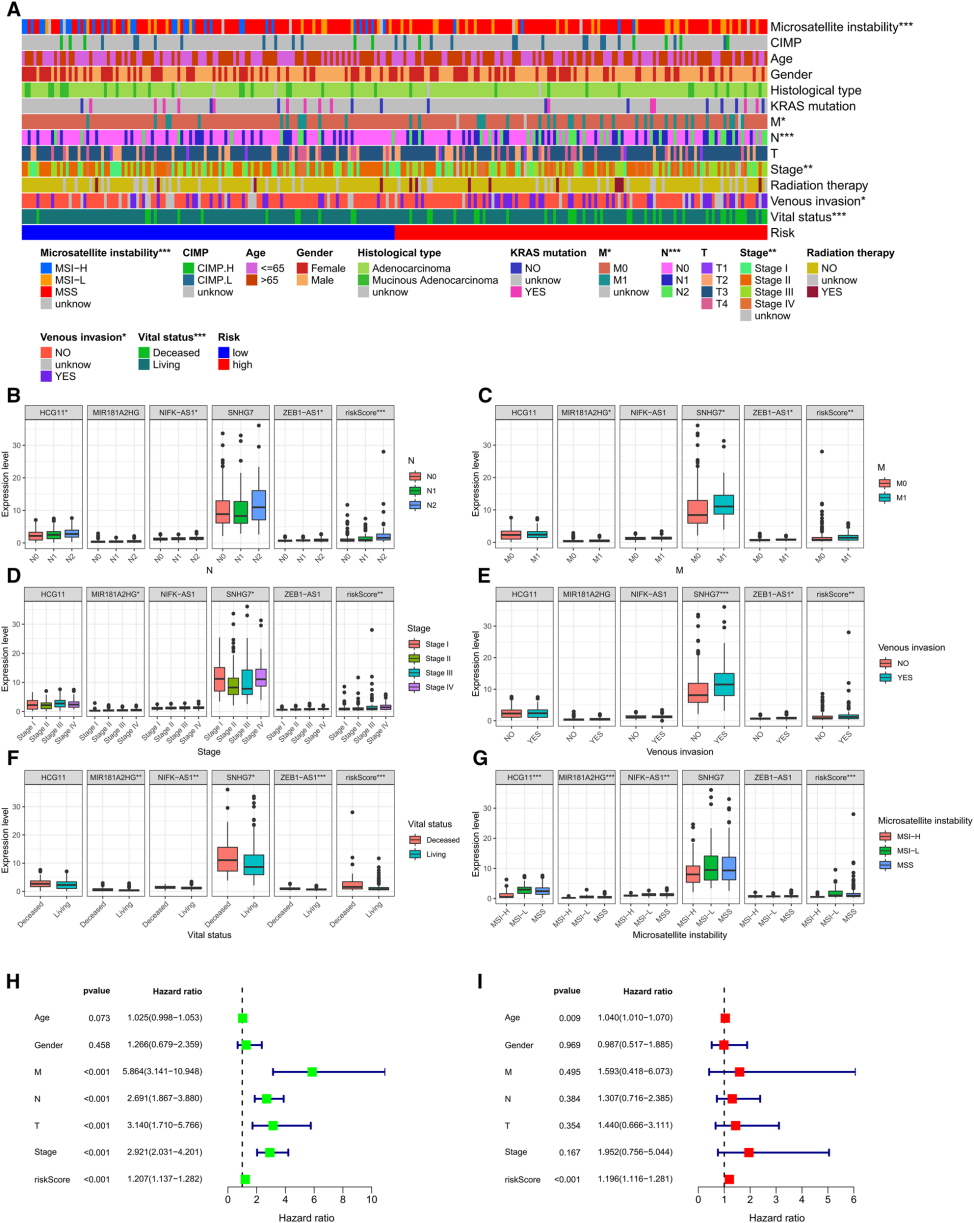
The correlation between the risk signature and clinical parameters. (**A**) The band diagram and box plots showed that (**B**) N classification, (**C**) M classification, (**D**) AJCC tumor stage, (**E**) Venous invasion, (**F**) Vital status, and (**G**) Microsatellite instability were significantly associated with the risk score. (**H**) Univariate Cox regression analysis revealed that AJCC tumor stage, T classification, N classification, M classification and risk score were statistically different. (**I**) Multivariate Cox regression analysis demonstrated that only age and risk score were independent prognostic predictors. *P < 0.05; **P < 0.01; ***P < 0.001.

We assessed the association between the signature and clinical parameters to further confirm the clinical values of the immune-related lncRNA signature. As shown in the band diagram (Fig. 4A), M classification, N classification, AJCC tumor stage, Microsatellite instability, Venous invasion and Vital status were different in a significant manner between the low- and high-risk groups, and box plots revealed that the risk score was roughly increased with N classification (Fig. 4B), M classification (Fig. 4C), AJCC tumor stage (Fig. 4D), and Venous invasion (Fig. 4E). Figure 4F showed that the risk score was elevated in the patient who was dead. Besides, CRC patients with microsatellite instability in MSS/MSI-L showed higher risk scores than those with MSI-H (Fig. 4G). In general, groups with aggressive clinicopathological characteristics were accompanied by higher risk scores, and genes in the signature were also significantly correlated with these clinicopathological characteristics. There were no significant correlations between the risk score and other clinical parameters (Supplementary Fig. 1). Then, univariate Cox regression analysis found that AJCC tumor stage (*P* < 0.001, HR 2.921, 95% CI [2.031–4.201]), T classification (*P* < 0.001, HR 3.140, 95% CI [1.710–5.766]), M classification (*P* < 0.001, HR 5.864, 95% CI [3.141–10.948]), N classification (*P* < 0.001, HR 2.691, 95% CI [1.867–3.880]), and risk score (*P* < 0.001, HR 1.207, 95% CI [1.137–1.282]) were considered statistically significant (Fig. 4H), whereas in the multivariate Cox analysis, only the age (*P* = 0.009, HR 1.040, 95% CI [1.010–1.070]) and risk score (*P* < 0.001, HR 1.196, 95% CI [1.116–1.281]) were revealed as independent prognostic predictors (Fig. 4I). This immune-related lncRNA signature could serve as a prognostic factor for CRC independent of clinicopathological factors.

### Immune-related lncRNA-mRNA co-expression network analysis

**Figure 5:**
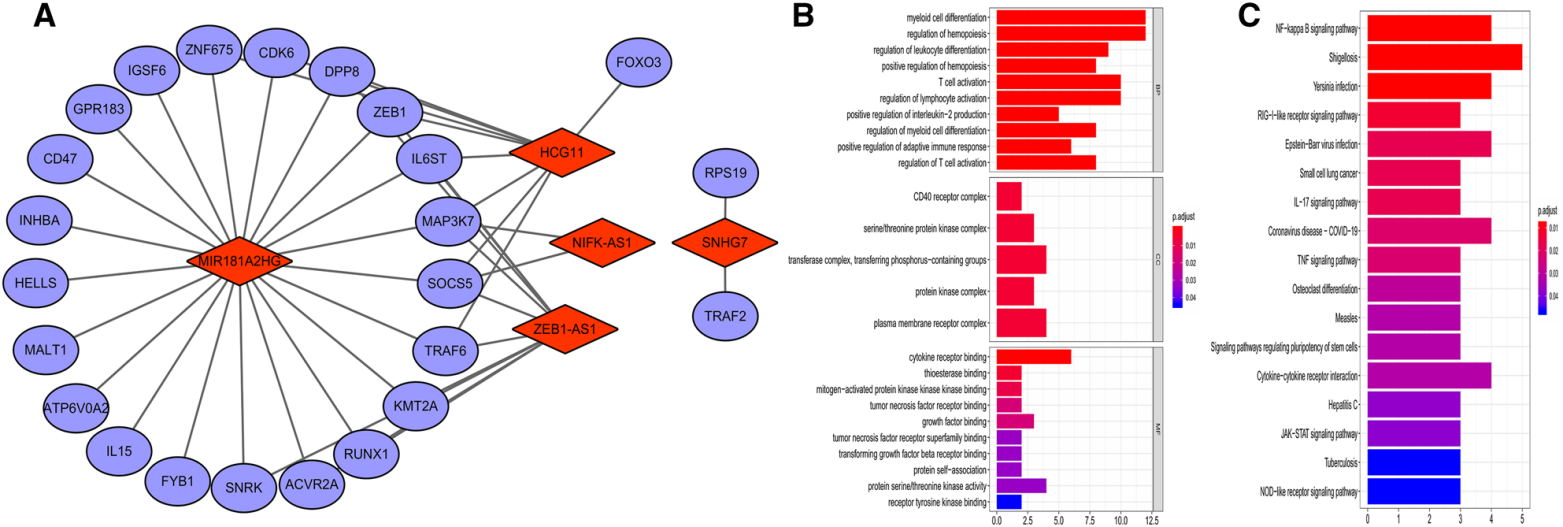
The co-expression network and functional enrichment analysis of the immune-related lncRNA signature. (**A**) The co-expression network between the immune-related genes and prognostic lncRNAs in CRC. (**B**) GO functional enrichment and (**C**) KEGG pathway enrichment analyses of the co-expression network.

Based on the correlation coefficient threshold > 0.5, and the corresponding *P* < 0.001, we found that these five immune-related lncRNAs had an obvious correlation with 24 immune-related mRNAs, and the lncRNA-mRNA co-expression relationship network was constructed and visualized by using Cytoscape software (Fig. 5A). Subsequently, GO enrichment analysis showed that the mRNAs co-expressed with the immune-related lncRNA signature mainly enriched in immunomodulatory pathways, such as myeloid cell differentiation, regulation of hemopoiesis, and regulation of leukocyte differentiation (Fig. 5B). KEGG pathway enrichment analysis showed these mRNAs mainly enriched in NF-κB signaling pathway, RIG-I-like receptor signaling pathway, IL-17 signaling pathway, and TNF signaling pathway (Fig. 5C).

### Construction of the prognostic nomogram

**Figure 6:**
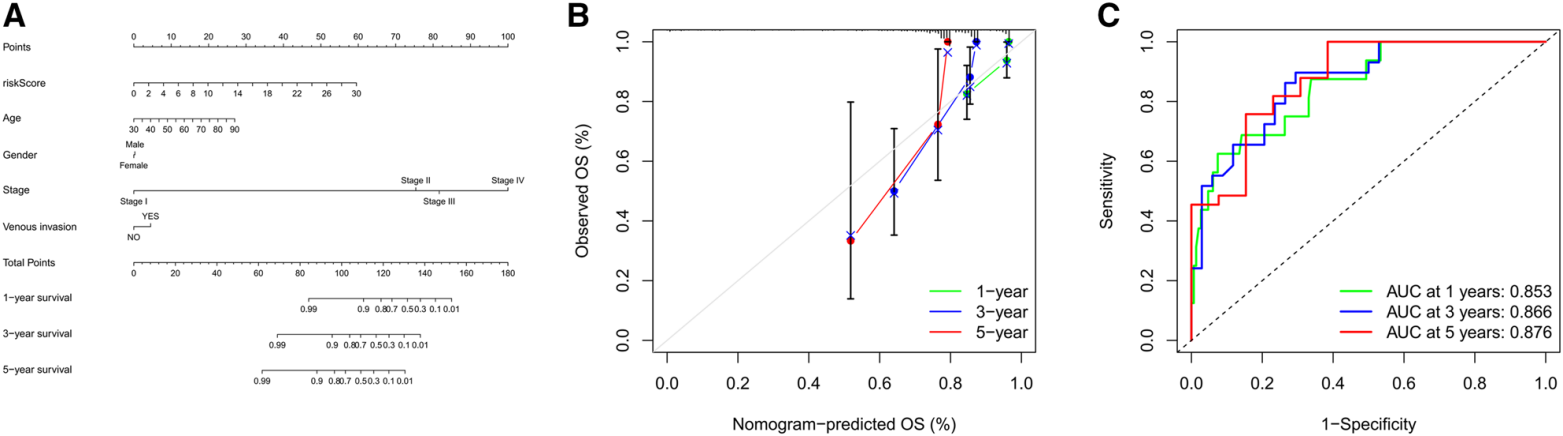
Construction of the prognostic nomogram. (**A**) Construction of the nomogram based on multivariate Cox regression models. (**B**) The 1-, 3-, and 5-years calibration plots for evaluating the agreement between the predicted and the actual prognosis for the prognostic signature. (**C**) The areas under the ROC curve about 1-, 3-, and 5-years of the nomogram.

Nomograms are usually applied to predict patient prognosis based on the score reflecting the values of several prognostic factors^30^. Therefore, we constructed a nomogram by integrating risk score and other clinicopathologic risk factors to better estimate the probability of survival at 1-, 3- and 5-years (Fig. 6A). The calibration plot showed a satisfactory agreement between predictive and actual values at the probabilities of 1-, 3- and 5-years survival (Fig. 6B). In addition, the AUC of 1-, 3- and 5-years survival for the nomogram were 0.853, 0.866, and 0.876, respectively (Fig. 6C). These findings indicated that the nomogram is precise and reliable in predicting the prognosis of CRC patients.

### Analysis of immune status between low- and high-risk groups

**Figure 7:**
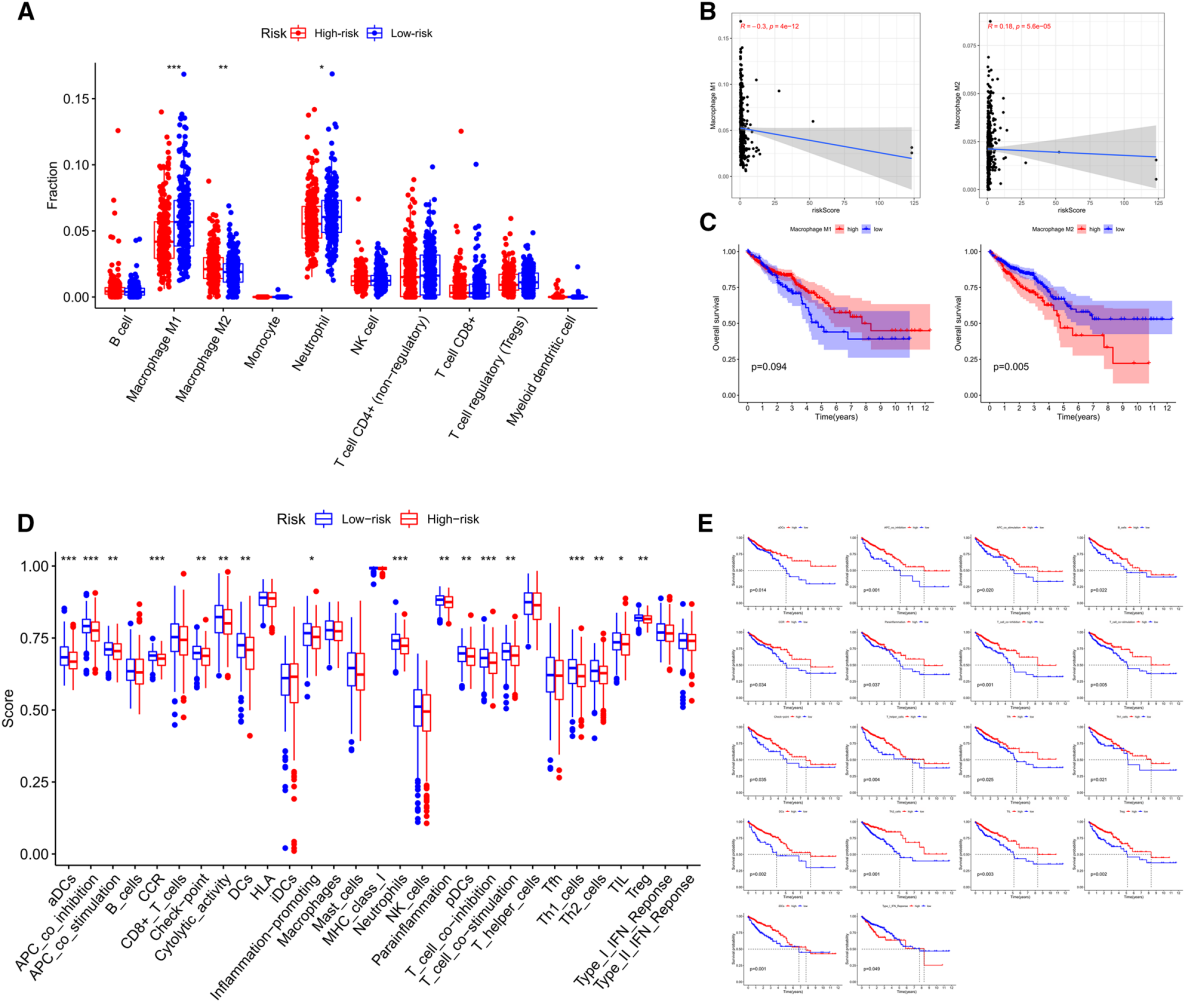
The correlation between the risk signature and immune status. (**A**) Relationship between the risk signature and 10 kinds of TIICs based on QUANTISEQ algorithm. (**B**) Estimation of the coefficients for risk score with the abundances of Macrophage M1 and Macrophage M2 based on Spearman correlation analysis. (**C**) Prognostic value of Macrophage M1 and Macrophage M2 in CRC patients. (**D**) Comparisons of immune functions in different risk groups. (**E**) Prognostic value of distinct immune functions in CRC patients.

QUANTISEQ algorithm was used to explore the ability of the immune-related lncRNA signature to predict the TIICs in CRC. As shown in Fig. 7A, High infiltration levels of Macrophages M1 and Neutrophil were detected in the low-risk group, while Macrophages M2 were highly infiltrated in the high-risk group. Spearman correlation analysis revealed that Macrophages M1 had a negative correlation with the risk scores of the patients, whereas Macrophages M2 had a positive correlation (Fig. 7B). We further analyzed the prognostic values of these TIICs and found that Macrophages M2 but not Macrophages M1 were significantly correlated with the prognosis of CRC patients (Fig. 7C). Interestingly, ssGSEA results further confirmed that immune functions such as T cell stimulation, inflammation promoting, and cytolytic activity were all significantly enhanced in the low-risk group (Fig. 7D). Survival analysis found that most of the immune function terms were correlated with increased survival time in CRC patients (Fig. 7E). To sum up, these investigations suggested that the low-risk group had elevated immune activity, which might contribute to anti-tumor effects and thereby improving the prognosis of CRC patients.

### Correlation between risk score and TMB

**Figure 8:**
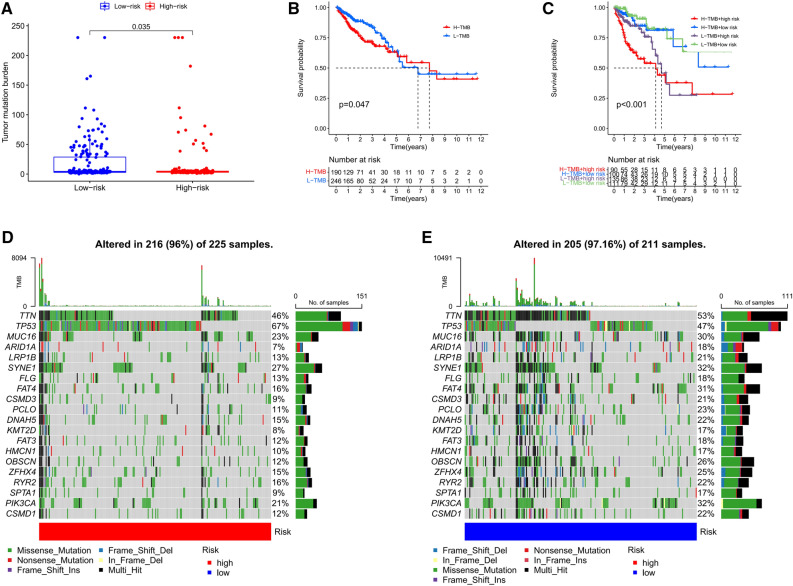
The correlation between the risk signature and TMB. (**A**) The TMB of low-risk group was significantly higher than that of high-risk group. (**B**) Kaplan–Meier curves of overall survival in different TMB subgroups. (**C**) Kaplan–Meier curves of overall survival stratified by both TMB and risk score. The waterfall diagram showed the top 20 driver genes exhibiting the highest mutation frequency in (**D**) high- and (**E**) low-risk groups.

We analyzed the difference in TMB between low- and high-risk groups and found that the levels of TMB in the low-risk group were significantly higher than that in the high-risk group (Fig. 8A). Then, we determined the prognostic value of TMB in CRC patients. As indicated from the results, TMB was a risk factor for the OS of CRC patients (Fig. 8B). We further assessed the effect of TMB and risk score on the prognosis of CRC patients considering their synergistic effect. As shown in Fig. 8C, the predictive ability of risk score was independent of TMB status. The survival difference between low- and high-risk groups was significant in both low- and high-TMB groups. In sum, the risk score serves as a predictive marker for OS of CRC patients that is independent of TMB and can effectively predict TMB status. Besides, we further evaluated the differences in mutation information for each gene between low- and high-risk groups and plotted the top 20 genes with the highest mutation frequency. As demonstrated in the waterfall diagram, mutation frequencies of these genes in the low-risk group were significantly higher than that in the high-risk group (Fig. 8D,E). Among them, TP53 was one of the most critical tumor suppressor gene that exhibiting higher mutation frequency in the high- than in the low-risk group.

### Immune-related lncRNA signature was significantly correlated with TIDE score in CRC samples

**Figure 9:**
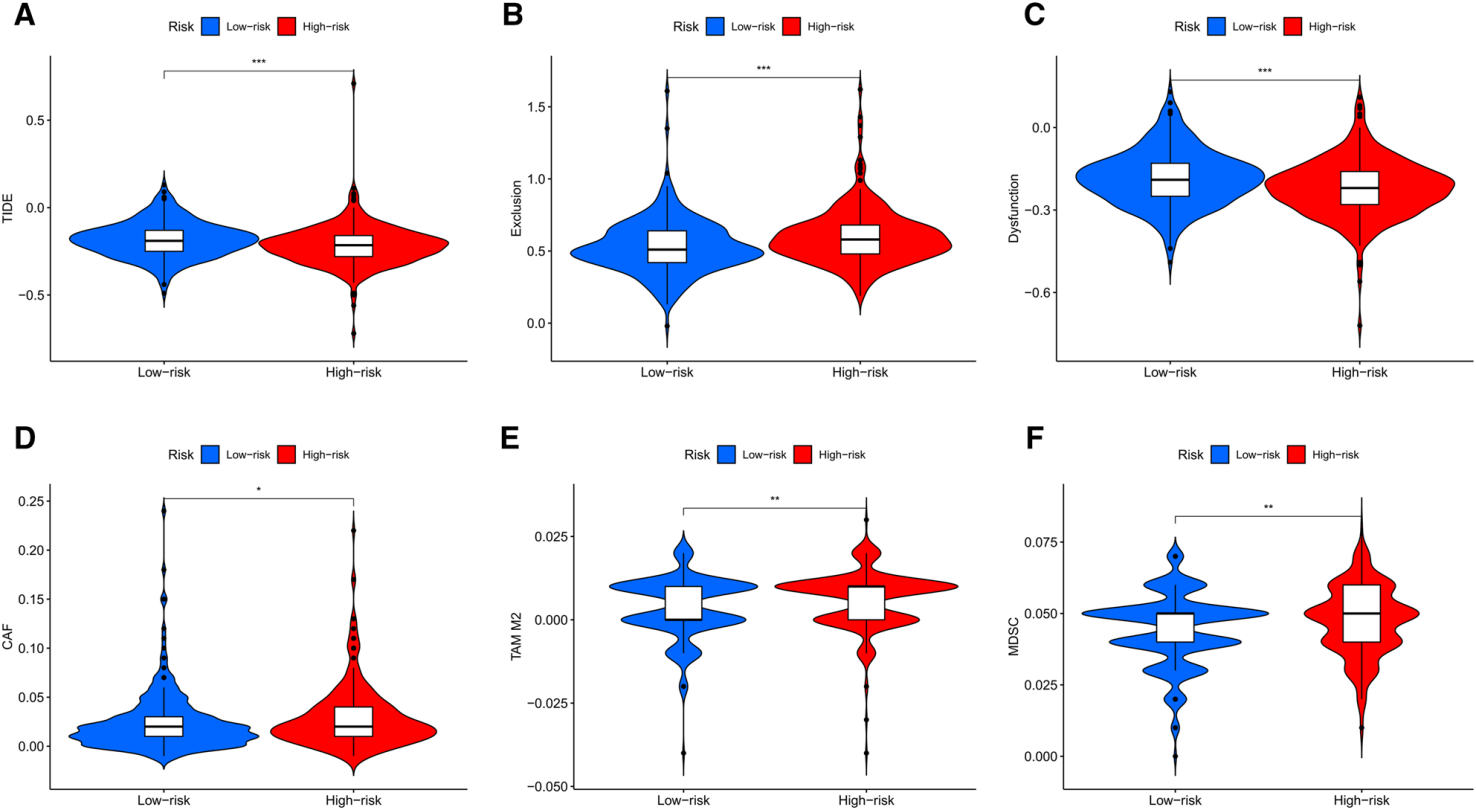
The correlation between the risk signature and T cell function. (**A**) Scores of TIDE, (**B**) T-cell exclusion and (**C**) dysfunction in different risk groups. (**D**) Infiltration level of CAF, (**E**) TAM M2, and (**F**) MDSC in different risk groups.

We applied TIDE to evaluate the potential treatment sensitivity of immunotherapy in low- and high-risk groups. As indicated from the results, the low-risk group had a higher TIDE score compared to the high-risk group (Fig. 9A). Besides, we found that the low-risk group had a lower T-cell exclusion score, but a lower T-cell dysfunction score (Fig. 9B,C). We further analyzed the differences in immunosuppressive TIICs, including cancer associated fibroblast (CAF), tumor associated macrophages M2 (TAM M2), and myeloid derived suppressive cell (MDSC) between low- and high-risk groups and found that all of them were highly infiltrated in the high-risk group (Fig. 9D–F). These results demonstrate that the immune-related lncRNA signature performs well in predicting the potential sensitivity of immunotherapy in the CRC samples.

## Discussion

Colorectal cancer, which causes severe health burdens worldwide, is the most frequently diagnosed malignant tumor of the digestive system. Although surgical resection has been proven to be the only curable therapy for CRC patients diagnosed at the localized stage, the prognosis of patients diagnosed at the advanced stage remains largely unsatisfactory^31^. Accumulating studies have indicated that dysregulation of the immune system is involved in the initial and progression of malignant tumor and may be one of the factors affecting the poor outcome of cancer^32,33^. In recent years, with the development of molecular biology and a greater understanding of mechanisms underlying the immune system, effective anti-tumor immunotherapy is now a reality^34^. Emerging immunotherapy methods such as immune checkpoint inhibitors (ICIs) have developed rapidly in tumor treatment and achieved a certain therapeutic effect^35^. It is now well-established that patients with mismatch-repair-deficient (dMMR): microsatellite instability-high (MSI-H) tumors tend to be more sensitive to anti-tumor immunotherapy methods such as ICIs^36^. However, for the vast majority (~ 85%) of CRC patients whose tumors are MMR-proficient (pMMR): microsatellite stable (MSS), immunotherapy currently provides little benefit^36,37^. Therefore, identifying novel biomarkers for immunotherapy is needed.

Previous studies have revealed that lncRNA serves as an important regulator of the immune response in multiple cancer types. For example, lncRNA *SATB2-AS1* has been reported to regulate TH1-type chemokines expression and immune cell density in CRC, thereby inhibiting tumor metastasis^38^. A recent study reported that lncRNA *MALAT1* promoted tumorigenesis and immune escape of diffuse large B cell lymphoma by sponging miR-195 to induce apoptosis of CD8+ T cells^39^. *GATA3-AS1* prevented the PD-L1 protein against ubiquitination through the *miR-676-3p*/*COPS5* axis, thereby facilitating immune escape in triple-negative breast cancer^40^. These findings suggested that immune-related lncRNAs may be better prognostic biomarkers and serve as potential targets for anti-cancer therapy.

In this study, we constructed a survival-related signature comprised of five immune-related lncRNAs (*HCG11, MIR181A2HG, NIFK-AS1, SNHG7,* and *ZEB1-AS1*) to predict the prognosis of CRC patients. Among these five candidate lncRNAs, *SNHG7* has been reported to be expressed at high levels in CRC samples compared to normal control^41^. *SNHG7* promoted proliferation and metastasis, and inhibited apoptosis of CRC cells via sponging *miR-34a* to regulate the PI3K/Akt/mTOR pathway^42^. It was reported that *ZEB1-AS1* expression was significantly higher in CRC tissues than in adjacent normal tissues, and patients with high *ZEB1-AS1* expression showed poorer prognosis than those with low expression^43^. In addition, *ZEB1-AS1* activated the Wnt/β-catenin signaling pathway by sponging miR-181-5p, thereby promoting the proliferation of CRC cells in vitro^44^. LncRNA *HCG11* has been experimentally demonstrated to facilitate the proliferation and migration of gastric cancer cells through the *miR-1276*/*CTNNB1*/Wnt pathway^45^. *NIFK-AS1* was reported to function as a sponge for miR-146a to suppress the M2-like polarization of macrophages, thereby inhibiting the estrogen-induced proliferation, migration, and invasion of endometrial cancer cells^46^. A recent study has identified *MIR181A2HG* as a reliable component of an immune-related signature to predict the OS of patients with bladder cancer^47^. Given that these five lncRNAs serve vital roles in tumor progression, our study comprehensively considered their expression patterns and combined them to develop a novel prognostic signature in human CRC. Subsequently, survival analysis revealed that this immune-related lncRNA signature could distinguish patients with favorable prognosis from those with poor prognosis. Thus, our signature can be used to predict the prognosis of CRC patients.

Functional enrichment analysis revealed that the immune-related lncRNA signature was mainly enriched in immunomodulatory pathways. Furthermore, KEGG enrichment analysis also revealed that the novel signature involved in various classical tumor-related pathways, which participate in the initial and progression of CRC, including NF-κB signaling^48^, TNF signaling^49^, RIG-I-like receptor signaling^50^, IL-17 signaling^51^ and JAK/STAT signaling^52^. Therefore, these results above suggested that the novel signature may participate in the progression of CRC through multiple signaling pathways.

Studies have indicated that the initial and progression of CRC involves many aspects of immunodeficiency, and dysfunction of TIICs may affect the efficiency of immunotherapy^53^. The composition of TIICs within CRC tumors contained immunostimulatory and immunosuppressive components. The former included CD8+ T cells, dendritic cells, and natural killer cells, which are involved in the control of tumor growth, whereas the latter included CAFs, TAMs, and MDSCs, which promote tumor growth and metastasis^54,55^. During the progression of tumor, immunosuppressive TIICs hold the dominant position in TME and exert their oncogenic effects by inhibiting T cell functions^56^. CAFs modulate cancer growth and metastasis through secreting growth factors and re-modeling of the extracellular matrix^57^. In CRC, the expression intensity of CAFs was found to be increased at the invasive borders of the tumor, and high intensity of CAFs was significantly correlated with advanced disease stage^58^. Moreover, previous studies have indicated that CAFs were able to affect sensitivity to oxaliplatin and 5-fluorouracil in CRC cells^59^. In addition to CAFs, TAMs also play a significant role in regulating immune function in TME. According to the environmental stimuli, TAMs are often thought to polarize towards a pro-inflammatory “M1” or anti-inflammatory “M2” state^60^. In CRC, M1 TAMs exert anti-cancer effects by secreting pro-inflammatory cytokines such as TNF-α and IL-1, and increased densities of M1 TAMs are linked with a favorable clinical outcome^61^. Conversely, M2 TAMs exert tumor-supporting effects by secreting immunosuppressive cytokines such as IL-10, and high abundances of M2 TAMs are associated with a poor prognosis^62^. MDSCs are a heterogeneous population of immature myeloid cells which can be found in many types of tumors. MDSCs have been reported to promote the metastasis of CRC by suppressing the activity of anti-tumor T cells and producing pro-tumorigenic factors^63^. In our study, high densities of CAFs, M2 TAMs, and MDSCs were detected in the high-risk group, while the infiltration fractions of M1 TAMs were higher in the low-risk group. These results indicated that the lncRNA signature could predict the immune characteristics of CRC, and patients in the high-risk group infiltrated with more immunosuppressive TIICs compared to those in the low-risk group. We further analyzed the correlation between the lncRNA signature and TMB, an emerging biomarker for sensitivity to immunotherapies in multiple types of cancer independent of microsatellite status or immune checkpoint expression^64^. Tumors with high TMB are thought to harbor enhanced immunogenicity because of increased neoantigen burden, and therefore more likely to benefit from immunotherapy^65^. Interestingly, our study also found that the levels of TMB in the low-risk group were significantly higher than that in the high-risk group. In sum, the results above suggested that the immune-related lncRNA signature might be a promising reference for CRC immunotherapy.

Inevitably, several limitations that existed in the present study should be pointed out. First and foremost, due to the lack of useful datasets containing lncRNA expression profiles and follow-up information of CRC in other public databases, the robustness of the immune-related lncRNA signature was validated in only one external cohort, multicenter perspective study will be performed to enhance the reliability of the signature in the future. Second, during the screening of candidate immure-related lncRNA, we took its prognostic value into consideration, which might omit some potentially valuable information and ultimately reduced the performance of the signature. Third, functional experiments should be conducted to further explore the functional roles of these immune-related lncRNAs in human CRC.

## Conclusions

In summary, the present study constructed and validated an immune-related five-lncRNA signature to predict the prognosis and immune characteristics of CRC patients based TCGA and GEO projects. There are significant differences in the clinical outcome and immune status of patients between low- and high-risk groups. This signature may provide new insights into the prognostic evaluation and individualized treatment for CRC patients.

## Supplementary Information

Supplementary Information.

Supplementary Figure 1.

## Data Availability

Thedatasetsgenerated and/or analyzed during the current study are available in The Cancer Genome Atlas (TCGA, https://www.cancer.gov/tcga), UCSC Xena (http://xena.ucsc.edu/), and Gene Expression Omnibus (GEO, https://www.ncbi.nlm.nih.gov/geo/) projects.

## Abbreviations

AJCC: American Joint Committee on Cancer
AUC: Area under the ROC curve
BP: Biological process
CC: Cell components
CRC: Colorectal cancer
dMMR: Mismatch-repair-deficient
GEO: Gene Expression Omnibus
lncRNA: Long non-coding RNA
TCGA: The Cancer Genome Atlas
MSigDB: Molecular Signatures Database
FPKM: Fragments Per Kilobase Million
ROC: Receiver operating characteristic
HR: Hazard ratio
C-index: Index of concordance
GO: Gene Ontology
MF: Molecular function
KEGG: Kyoto Encyclopedia of Genes and Genomes
MSI-H: Microsatellite instability-high
pMMR: MMR-proficient
MSS: Microsatellite stable
