# Supplementary material for: Identification of prognostic immune-related lncRNA signature predicting the overall survival for colorectal cancer
Source: Sci Rep. 2023 Jan 24;13:1333. doi: 10.1038/s41598-023-28305-9 (PMC9873726; doi:10.1038/s41598-023-28305-9)
Supplement: Supplementary file 1 — Supplementary Information. [file 41598_2023_28305_MOESM1_ESM.pdf]

KEGG Database Project
Kanehisa Laboratories
www.kanehisa.jp

Ref: 221754

Permission is granted to Scientific Reports of Springer Nature Ltd to publish both in print and digital under the CC BY 4.0 open access license the following KEGG pathway map images in the article "Identification of prognostic immune-related lncRNA signature predicting the overall survival for colorectal cancer" written by Qingqiang Yang and colleagues:

- TNF signaling pathway (map04668)
- IL-17 signaling pathway (map04657)
- RIG-I-like receptor signaling pathway (map04622)
- NF-kappa B signaling pathway (map04064)

subject to the condition that the original source is acknowledged by citing at least one KEGG paper.

Permission granted: Naomi Takahashi

Naomi Takahashi, Kanehisa Laboratories

Date: 8 December 2022

Copyright holder: Kanehisa Laboratories
