# Supplementary figures and images for: Identification of prognostic immune-related lncRNA signature predicting the overall survival for colorectal cancer
Source: Sci Rep. 2023 Jan 24;13:1333. doi: 10.1038/s41598-023-28305-9 (PMC9873726; doi:10.1038/s41598-023-28305-9)

**Supplementary Figure 1**. The correlation between the risk signature and clinical parameters.


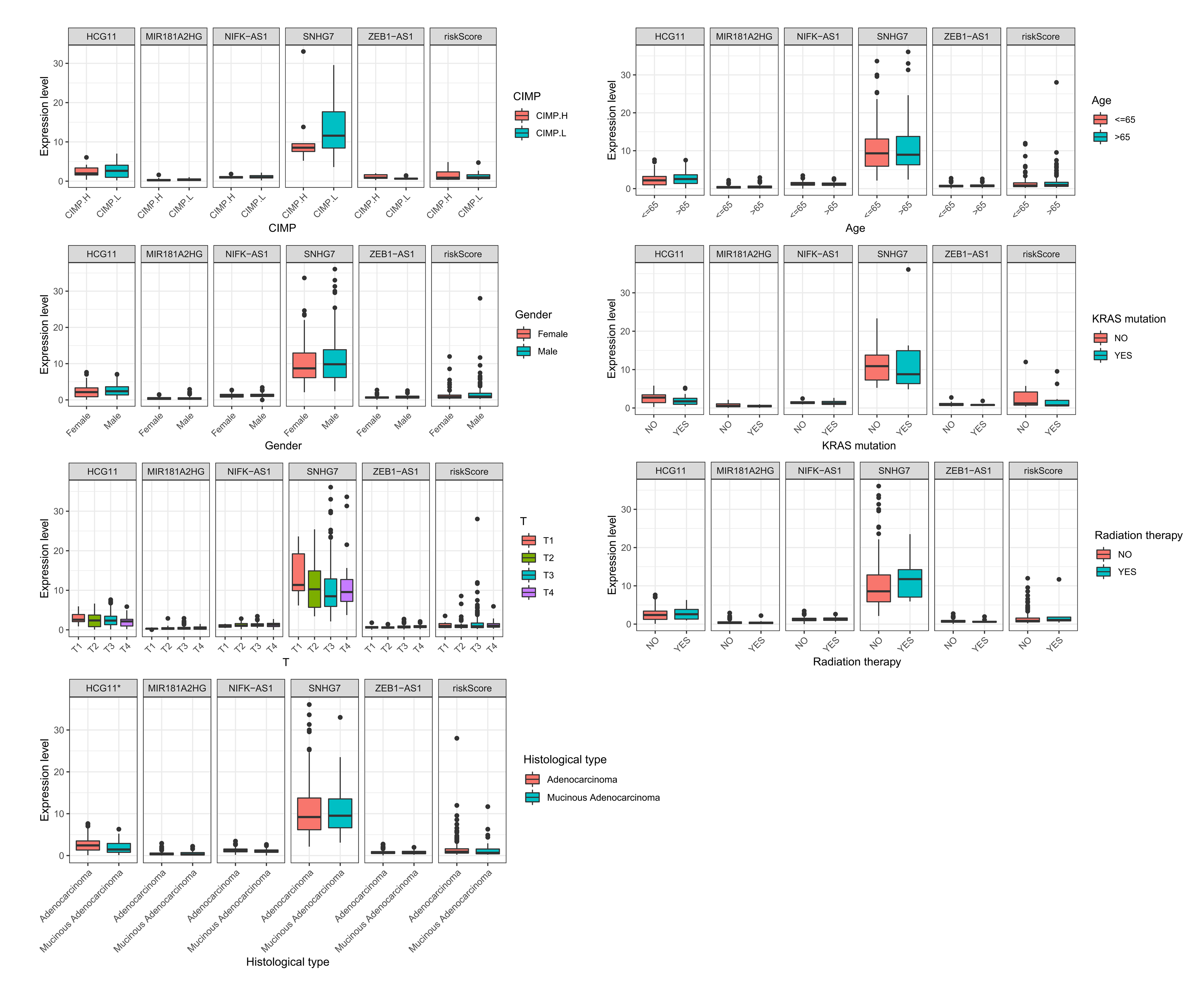

Supplement: Supplementary file 2 — Supplementary Figure 1. [file 41598_2023_28305_MOESM2_ESM.docx]
